# Detection of adverse events in older adults undergoing surgery using the IHI global trigger tool within the SURGE-Ahead project

**DOI:** 10.1186/s12877-025-06833-5

**Published:** 2025-12-17

**Authors:** Jessica Wolf, Filippo Maria Verri, Thomas D. Kocar, Dhayana Dallmeier, Raffael Cintean, Florian Gebhard, Christian Bolenz, Nuh Rahbari, Annabel S. Mueller-Stierlin, Michael Denkinger, Christoph Leinert

**Affiliations:** 1 Institute for Geriatric Research Ulm, Ulm University Medical Center, Ulm, Germany; 2 Geriatric Center Ulm, AGAPLESION Bethesda Ulm, Ulm, Germany; 3 Department of Epidemiology, Boston University School of Public Health, Boston, USA; 4 Department for Orthopedic Trauma, Ulm University Medical Center, Ulm, Germany; 5 Department of Urology, Ulm University Medical Center, Ulm, Germany; 6 Department of General and Visceral Surgery, Ulm University Medical Center, Ulm, Germany; 7 Institute for Epidemiology and Medical Biometry, Ulm University, Ulm, Germany

**Keywords:** IHI global trigger tool (GTT), Adverse event, Geriatrics, Surgery, Geriatric co-management

## Abstract

**Background:**

The aim of this study was to identify the number, type and severity of adverse events (AE) and the factors associated with the (non)-occurrence of AE in older surgical inpatients within the SURGE-Ahead project.

**Methods:**

A retrospective chart review within a larger prospective observational study was conducted in three surgical departments of Ulm University Medical Center (trauma surgery, general surgery, urology). Patients undergoing inpatient elective or emergency surgery aged ≥ 70 years with an Identification of Seniors at Risk (ISAR) score ≥ 2 were included. A modified version of the Institute for Healthcare Improvement (IHI) Global Trigger tool (GTT) adapted to the geriatric cohort was utilized to identify AE during hospitalization and within 30 days after discharge. Binary logistic regression was conducted to identify factors associated with an adverse event-free treatment course.

**Results:**

Of 174 included participants, 131 (75.3%) had at least one AE. In total 348 AEs were identified, which can be expressed as 155.9 AE per 1000 patient days or 200.0 AE per 100 admissions. In most cases the AE resulted in temporary harm requiring intervention (58.9%). Intra-/postoperative AE (43.8%) and nosocomial infections (18.9%) occurred most frequently. Younger age, shorter operation time and absence of frailty were significantly associated with an AE-free treatment course (AUC 0.81).

**Conclusion:**

A notable number of AE were detected in the study population. The addition of the triggers „new Impairment of cognition/vigilance, fluctuating confusion” and “electrolyte disturbances” enhanced the efficacy of the IHI GTT in the geriatric population. Age, frailty status and operation time might predict the need for geriatric co-management.

**Trial registration:**

German clinical trials registry (Deutsches Register für klinische Studien, DRKS00030684), registered on 21st November 2022.

**Supplementary Information:**

The online version contains supplementary material available at 10.1186/s12877-025-06833-5.

## Introduction

The documentation of adverse events (AE) in hospitals is crucial for improving patient safety and healthcare quality [1]. According to the World Health Organization (WHO) an AE is defined as an unintentional harm or injury related to medical care [1, 2]. Hospitalized patients, particularly older adults, are vulnerable to AEs, which are often preventable and can lead to significant morbidity and mortality [3]. Existing studies estimate that approximately 10% of hospitalized patients experience an AE [4], with rates potentially higher in complex geriatric populations [5]. A systematic review has investigated the incidence and types of AE among adult patients during hospitalization: in eight studies with a total of 74 485 patient records from the USA, UK, Australia, New Zealand and Canada nearly one in ten patients experienced an AE during hospitalization [3]. The Institute for Healthcare Improvement (IHI) Global Trigger Tool (GTT) is a widely used method for identifying AEs [1]. A study compared the IHI GTT with other methods of AE detection, such as voluntary reporting systems and the “Agency for Healthcare Research and Quality’s Patient Safety Indicators”, which are sets of metrics to identify risks to patient safety [6, 7]. In total, these three methods identified 393 AE in 795 patient records. Results showed that the IHI GTT was able to identify 90.1% of all AE, the voluntary reporting systems 1.0% and the Patient Safety Indicators 8.99% of all AE, demonstrating the strong detection capabilities of the IHI GTT. Although precisely quantifying the total number of AEs presents a significant challenge, the study demonstrated that the IHI GTT was able to detect ten times more AEs compared to other tools [6]. However, the IHI GTT possesses inherent limitations when applied to geriatric patients, owing to the absence of triggers specifically linked to common geriatric AEs such as delirium [8]. In the present study, we adapted the IHI GTT with geriatric-specific triggers to assess the incidence and types of AEs in older surgical inpatients. The goal is to identify the prevalence of AEs in this vulnerable population and determine associated risk factors to improve the quality of care.

## Methods

The retrospective data collection was conducted within a prospective observational cohort study in the “Supporting SURgery with GEriatric co-management and AI” (SURGE-Ahead) project.

### Study population

During a 12-month recruitment phase a total of 178 participants of three surgical departments (trauma surgery (TRA): *n* = 131, general surgery (GEN): *n* = 23), urology (URO): *n* = 24) of the Ulm University Medical Center were enrolled after written consent. Inclusion criteria were age ≥ 70 years, Identification of Seniors at Risk (ISAR) score ≥ 2 at the time of admission, no participation in another study, and any kind of surgical intervention [9]. ISAR score was used to include especially older participants presenting with a geriatric care need on admission. 174 cases were included in the analysis after removal of four dropouts. Dropout reasons were self-withdrawal from participation due to loss of interest, and death before surgery.

### Data collection

On admission data was collected on living situation (living site, living alone/with spouse), pictogram-based Clinical Frailty Scale (CFS) [10, 11] using 2 weeks before admission as a reference, history of dementia and Barthel Index (BI preop) and 2 weeks prior to presentation (BI prior) [12]. Details on the inpatient stay such as length of hospital stay (LOS), length of stay in the intensive care unit (LOS ICU), operation time (cut-to-suture time), type of admission (elective versus emergency) and the classification system of the American Society of Anesthesiologists (ASA) Score were collected from the Hospital Information System (HIS) [13]. For a complete description of the study, we refer to the previously published study protocol [14].

### Global trigger tool and AE classification

The IHI GTT is a comprehensive chart review consisting of two steps: (1) patient records are screened for a trigger (or clue) indicative for the occurrence of an AE. (2) if a trigger is identified the chart is reviewed in more depth to confirm or reject the suspected AE. In our study, triggers were screened from admission to discharge of the respective hospitalization. An AE present on admission was only included if related to previous medical or surgical care. The IHI GTT, originally containing 53 triggers, was adapted to the geriatric cohort and translated into German. An additional module “Geriatrics” with 7 triggers was added after initial review of the IHI GTT by the research team for typical triggers that may lead to AE in a vulnerable geriatric population (“New impairment of cognition/vigilance, fluctuating confusion”; “Vomiting”; “Change in weight > 2kg”; “Fever or hypothermia”; “Bacteriuria in urine culture”; “Rising CRP-level”; “Bedside watch”). To enhance systematical review for the identification of nosocomial infections the trigger “Healthcare-associated infection” and “Pneumonia onset” were replaced by following triggers: “Fever or hypothermia”, “Bacteriuria in urine culture” and “Rising CRP-level”. Perinatal module triggers were excluded. The trigger “Time in emergency department greater than 6 hours” was excluded because the exact length of stay in the emergency department could not be determined. Readmission to Ulm University Medical Center within a period of 30 days after discharge from hospital is a trigger in the IHI GTT. A total of 46 triggers were used in our adapted version and are described in Additional file 1. Confirmed AEs are then categorized by their severity of harm. The IHI GTT employs a classification system based on the National Coordinating Council for Medication Error Reporting and Prevention (NCC MERP) Index for categorizing medication errors [15]. This classification was adopted in the IHI GTT for the classification of AE. In the original version of the IHI GTT, the following AE (Category E to I) resulting in patient harm that led to a consequence are considered: Category E: Temporary patient harm requiring intervention, Category F: Temporary patient harm requiring initial or prolonged hospitalization, Category G: Permanent patient harm, Category H: Patient harm requiring intervention to sustain life, Category I: Patient death. Two additional categories were added in the version used in our study to include AE that did not lead to an intervention (Category C and D). Category C describes AE that required no additional monitoring, no intervention and no prolonged hospitalization. Category D includes AE that required additional monitoring but no intervention and no prolonged hospitalization (see Table 2). Types of AE were classified in twelve different groups: Intra-/postoperative AE, Drug side effect, Nursing care, Nosocomial infection, Neurological problems, Cardiac system, Pulmonary system, Renal system, Gastrointestinal system, Electrolyte disturbance, Allergic reaction, Other.,

Triggers and AE were recorded retrospectively for all included participants through a comprehensive chart review. In a first step the charts were reviewed for triggers and associated AEs as proposed by the IHI GTT training manual [16]. The chart reviewed included the discharge letter, patient charts including vital signs, medications and nursing documentation, the results of laboratory and other investigations (e.g. x-rays), surgeons notes and digital HIS documentation, that was provided by different members of the multidisciplinary team (e.g. nurses, surgeons, therapists). In parallel also AEs that were not associated with a trigger but were found during the review were recorded. The AEs were recorded by a physician (JW) under the supervision of an experienced geriatrician (CL), both trained for using the IHI GTT by means of the IHI training record [1]. Surgeons of the participating departments were consulted in unclear cases during analyses. In the original version of the IHI GTT the review should be done by three researchers, which was not possible in this study, with only two researchers being involved. However, the IHI GTT has been successfully adapted in this way in other studies before [17]. To ascertain data quality no time limit was set for the review of the participants’ records to enable a more detailed chart review.

### Statistical analysis

As recommended in the IHI GTT, AE per 1000 patient days, AE per 100 admissions and the percentage of patients with at least one AE were analyzed [1]. The positive predictive value (PPV) and its 95% CI was calculated based on the Wald method for each trigger indicating what proportion of the trigger was linked to an identified AE in the chart review [18]. Descriptive statistics were performed for the total population and different subpopulations (TRA, GEN, URO). Mann-Whitney-U-Test, Chi-Quadrat-Test, Fisher-Exact-Test or Fisher-Freeman-Halton-Test were performed to determine differences in patient characteristics between participants with an AE and no AE.

We performed a multivariable logistic regression for the identification of predictors of AE. Variable selection was done based on literature, domain knowledge, and group differences, avoiding multicollinearity. Age, frailty and operation time have been described as risk factors for AE in the literature [19–21]. In addition, the variables BI, level of care, LOS and LOS ICU were considered as potential predictors. Being part of the inclusion criteria as well as representing in part acute decline directly before admission, ISAR scores were not considered. LOS ICU was excluded as a predictor because it is often a consequence of, rather than a precursor to, AE. LOS was excluded due to its dependence on reimbursement rates. CFS, level of care and BI exhibited high collinearity, suggesting a strong inter-correlation between these three variables. Among these, CFS was selected as a predictor, being a multidimensional assessment with reference 2 weeks before admission compared to the ADL focused level of care and BI. For analysis, the age and operation time variables were z-transformed, the CFS scores binarized (cut-off: ≥ 5 = frail). No post-hoc power analysis was performed. Model adequacy was judged by events per variable [22].

All statistical data analyses were performed with SPSS (version 29.0.1.0). A *p*-value < 0.05 was considered statistically significant, but only in a descriptive way because of multiple testing procedures. Figures were created with Microsoft Office Excel 2016 or SPSS.

## Results

### General characteristics

A total of 174 cases were analyzed, of which 94 cases (55%) had a CFS score of 5 or more and were considered frail. The highest number of older people living with frailty was observed in the TRA (*n* = 81, 62,8%). The proportion of female participants in the TRA was greater than in the other departments (all: *n* = 100, 57.5%; TRA: *n* = 87, 67.4%). Admission types varied across the departments. Most participants from the TRA were admitted as an emergency (*n* = 92, 71.3%), whereas the GEN and URO primarily received elective admissions (*n* = 19, 82.6% and *n* = 22, 100%). The median length of stay in the intensive care unit (LOS ICU) was longest in the URO (2,0 (0,3–165) days). A total of 17 (13.6%) patients in the TRA had a known diagnosis of dementia, while none were identified in the other departments (Table 1).

**Table 1:** Characteristics of the overall study population and each department (“TRA”, “GEN”, “URO”)

Variable	Total group *n* = 174	TRA *n* = 129	GEN *n* = 23	URO *n* = 22
Age, years				
Median (Q1, Q3)	82 (75, 85)	82 (76, 86)	80 (74, 84)	78 (72, 83)
Sex				
Female, n (%)	100 (57.5)	87 (67.4)	7 (30.4)	6 (27.3)
Male, n (%)	74 (42.5)	42 (32.6)	16 (69.6)	16 (72.7)
Dementia				
Yes, *n* (%)	17 (10.1)	17 (13.6)	0	0
No, *n* (%)	152 (89.9)	108 (86.4)	22 (100.0)	22 (100.0)
Length of hospital stay (days)				
Median (Q1, Q3)	10 (7, 14)	11 (8, 14)	12 (8, 20)	7 (6, 12)
Operation time (min)				
Median (Q1, Q3)	68 (44, 133)	54 (36, 82)	179 (136, 218)	150 (108, 184)
Length of stay ICU^a^ (h)				
Median (Q1, Q3)	2 (1, 4)	2 (1, 2)	15 (3, 19)	18 (7, 20)
Admission type				
Elective, *n* (%)	78 (44.8)	37 (28.7)	19 (82.6)	22 (100)
Emergency, *n* (%)	96 (55.2)	92 (71.3)	4 (17.4)	0
Level of nursing care ^b^				
Yes, *n* (%)	81 (46.6)	67 (51.9)	7 (30.4)	7 (31.8)
No, *n* (%)	93 (53.4)	62 (48.1)	16 (69.6)	15 (68.2)
CFS ^c^				
non-frail (< 5), *n* (%)	77 (45.0)	48 (37.2)	16 (72.7)	13 (65.0)
frail (≥ 5), *n* (%)	94 (55.0)	81 (62.8)	6 (27.3)	7 (35.0)
ASA ^d^				
0, *n* (%)	2 (1.2)	2 (1.6)	0	0
1, *n* (%)	23 (13.3)	18 (14.1)	2 (8.7)	3 (13.6)
2, *n* (%)	132 (76.3)	98 (76.6)	17 (73.9)	17 (77.3)
3, *n* (%)	16 (9.2)	10 (7.8)	4 (17.4)	2 (9.1)
ISAR ^e^				
Median (Q1, Q3)	3 (2, 4)	3 (2, 4)	3 (2, 3)	2 (2, 3)
BI-prior ^f^				
< 50, *n* (%)	11 (6.3)	10 (7.8)	1 (4.3)	0
50–80, *n* (%)	39 (22.4)	32 (24.8)	3 (13.0)	4 (18.2)
> 80, *n* (%)	124 (71.3)	87 (67.4)	19 (82.6)	18 (81.8)
BI-preop>^g^				
< 50, *n* (%)	76 (43.7)	74 (57.4)	2 (8.7)	0
50–80, *n* (%)	45 (25.9)	38 (29.5)	3 (13)	4 (18.2)
> 80, *n* (%)	53 (30.5)	17 (13.2)	18 (78.3)	18 (81.8)
Living situation at home				
Living alone, *n* (%)	79 (45.4)	65 (50.4)	7 (30.4)	7 (31.8)
With partner/relatives, *n* (%)	93 (53.4)	62 (48.1)	16 (69.6)	15 (68.2)
With 24 h caregiver, *n* (%)	2 (1.1)	2 (1.6)	0	0
Living site				
House, *n* (%)	90 (51.7)	62 (48.1)	13 (56.5)	15 (68.2)
Apartment, *n* (%)	60 (34.5)	48 (37.2)	6 (26.1)	6 (27.3)
Assisted living, *n* (%)	6 (3.4)	5 (3.9)	0	1 (4.5)
Nursing home, *n* (%)	18 (10.3)	14 (10.9)	4 (17.4)	0
Adverse events (AE)				
Total AE, *n* (%)	348	231 (66.4)	63 (18.1)	54 (15.5)
AE/patient	2.0	1.8	2.7	2.4
AE/1000 patient days	155.9	134.5	200.6	270.0
AE/100 admissions	200.0	180.5	273.9	245.5
Participants with ≥ 1 AE, *n* (%)	131 (75.3)	91 (70.5)	21 (91.3)	19 (86.4)
AE without recorded trigger, *n* (%)	60 (100)	42 (70.0)	11 (18.3)	7 (11.7)

^a^*ICU* Intensive care unit, ^b^Level of nursing care in Germany: “Pflegegrad”, ^c^*CFS* Clinical frailty scale, ^d^*ASA* American Society of Anaesthesiologistˈs-risk classification, ^e^*ISAR* Identification of seniors at risk, ^f^*BI prior* Barthel-Index (BI), which refers to the time 2 weeks prior to hospital admission, ^g^*BI preop* refers to the time preoperatively

### Triggers

The chart review revealed 551 positive triggers. The PPV for an AE was calculated for each trigger. The most common triggers were “Decrease of greater than 25% in hemoglobin” (*n* = 62, 10.9%, PPV 95% (CI 89.2;100)), “Transfusion or use of blood products” (*n* = 58, 10.2%, PPV 81% (CI 71;91.1)) and “Admission to ICU” “(*n* = 50, 8.8%, PPV 42% (CI28.3;55.7)). Seven Triggers were identified only once (e.g. “Restraint use”) and ten not at all (e.g. “Flumazenil use”), in these cases we did not calculate any PPV or CI. Of 46 triggers identified, a total of 20 achieved a PPV > 50%. The number, PPV including CI and the number of AE associated with each trigger are described in detail in Additional file 2.

The modules with the most reported triggers accounted to Module C (Care) (*n* = 155, 27.3%), M (Medication) (*n* = 103, 18.2%), G (Geriatrics) (*n* = 160, 28.2%). In Module C “Transfusion of blood products” (*n* = 58, 10.2%) was the most frequent trigger. In Module M “Anti-emetic use” (*n* = 50, 8.8%) and in Module G “New impairment of cognition/vigilance” (*n* = 36, 6.3%) were identified as frequent triggers.

The comprehensive chart review took on average approx. 60–90 min per case with a minimum of 20 min and a maximum of 180 min per case.

### AE rates

As multiple triggers signaled the same AE, only 348 individual AE were observed. 131 out of 174 participants (75.3%) had at least one AE. A higher proportion of participants in GEN (*n* = 21, 91.3%) and URO (*n* = 19, 86.4%) experienced at least one AE, compared to TRA (*n* = 91, 70.5%) (Table 1). An average of 1.8 AE/participant in TRA, 2.7 AE/participant in GEN and 2.4 AE/participant in URO was found in the different departments. A total of 155.9 AE per 1000 patient days and 200 AE per 100 admissions were observed for the entire study population. Department-specific rates were as follows: TRA: 134.5 AE per 1000 patient days and 180.5 AE per 100 admissions, GEN: 200.6 AE per 1000 patient days and 273.9 AE per 100 admissions and URO: 270.9 AE per 1000 patient days and 245.5 AE per 100 admissions. Of the 348 identified AE, 36 (10.3%) were present at the time of admission. Of the 36 AE recorded at the time of admission, 23 (6.6%) AE were documented at initial admission, whereas 13 (3.7%) AE occurred during readmission within the 30-day period following discharge. 288 (82.8%) AE were associated with a trigger, 60 (17.2%) AE were identified without a trigger. Especially electrolyte disturbances (*n* = 25) and healthcare-associated infections (*n* = 15) were all identified without a trigger.

### Category of harm

The most frequent AE in all three departments were temporary harm to the participants requiring intervention (Category E, *n* = 205, 58.9%). AEs that were life-threatening (Category H, *n* = 11, 3.2%) or were associated with death (Category I, *n* = 9, 2.6%) were less common (Table 2).

The nine AE associated with death (TRA *n* = 4, GEN *n* = 3, URO *n* = 2) were extracted from three participants (1 in each department) that died in the study cohort. These cases were analyzed in more detail: In the first case (TRA) a peri-implant infection was treated, and the participant presented with decompensated heart failure, acute respiratory failure following aspiration, and acute kidney failure in the treatment course, contributing finally to the patient’s death. In the second case (GEN), the patient had a locally invasive neuroendocrine carcinoma of the stomach. Postoperatively, there were complications such as anastomotic leakage with gastrointestinal bleeding, bleeding from the splenic artery, and acute respiratory failure following hematemesis. Eventually, it was decided to pursue palliative care according to the presumed will of the patient upon discovering peritoneal carcinosis. In the third case (URO), a patient undergoing resection of a locally advanced bladder leiomyosarcoma developed a post-operative paralytic ileus, complicated by vomiting and aspiration. Subsequently, the patient experienced an acute cardiac arrest later and could not be resuscitated.

**Table 2:** The categorization of harm according to the NCC MERP Index, and the distribution in total and in the three departments

	Total (*n*,%)	TRA (*n*, %)^a^	GEN (*n*, %)^a^	URO (*n*, %)^a^
NCC MERP Category				
C - Temporary harm without monitoring	49 (14.1)	36 (15.6)	9 (14.3)	4 (7.4)
D - Temporary harm with monitoring	19 (5.5)	16 (6.9)	3 (4.8)	0 (0)
E - Temporary harm with intervention	205 (58.9)	133 (57.6)	32 (50.8)	40 (74.1)
F - Temporary harm with prolonged hospitalization	50 (14.4)	35 (15.2)	11 (17.5)	4 (7.4)
G - Permanent patient harm	5 (1.4)	2 (0.9)	2 (3.2)	1 (1.9)
H - Intervention to sustain life	11 (3.2)	5 (2.2)	3 (4.8)	3 (5.6)
I – Association to patients’ death	9 (2.6)	4 (1.7)	3 (4.8)	2 (3.7)
Total	348 (100)	231 (66.4)	63 (18.1)	54 (15.5)

^a^Percentages refer to the total number of AE in the department

### Type of AE

**Fig. 1:**
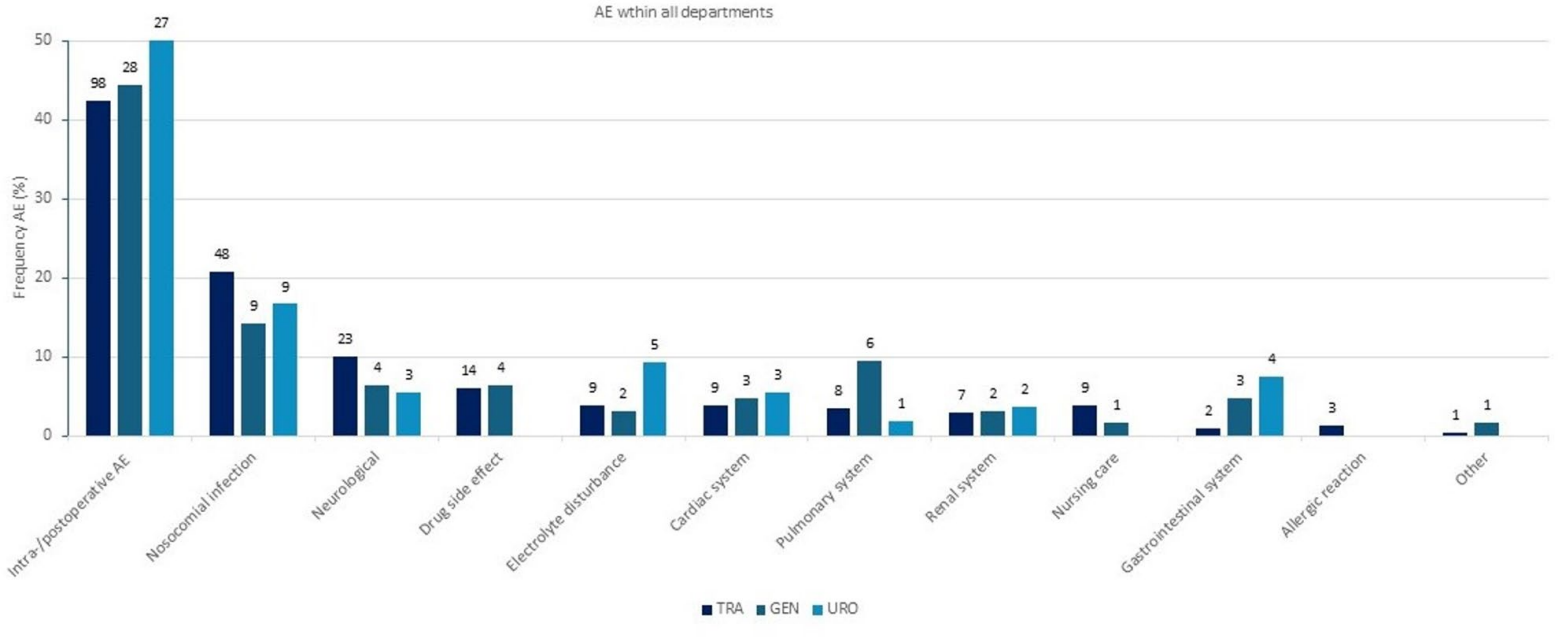
Types of AE and their distribution in the three departments (“TRA”, “GEN”, “URO”). Y axis: Frequency AE (%) among the three departments TRA, GEN and URO. Percentages refer to the total number of AE in the category. Numbers outside the columns show the number of AE (n) in each department

Intra/postoperative AE were identified as the most frequent type of AE (*n* = 153. 43.8%) (Fig. 1). Within this group “Postoperative anemia” (*n* = 56, 16.1%) and “Postoperative vomiting and nausea” (*n* = 34, 9.8%) occurred most frequently. Nosocomial infections (*n* = 66. 18.9%) and neurological AE (*n* = 30, 8.6%) were also common. Overall, the most prevalent type of infection was “Urinary tract infection” (*n* = 26, 7.5%) and “Delirium” (*n* = 28, 8.0%) the most common neurological AE. The distribution of the different types of AE across the departments is shown in detail in Additional file 3. Figure 1 provides an overview of the types of AE.

### Characteristics of participants with AE/no AE

For further analysis, the study population was divided into two groups: Participants who experienced at least one AE (*n* = 131, 75.3%) and those who did not experience an AE (*n* = 43, 24.7%) during hospitalization. Participants with at least one AE showed a significant higher LOS, LOS ICU and operation time. They were mostly older persons living with frailty and showed a significantly lower value of the BI prior to admission (BI-prior). No significant difference was found concerning participants undergoing elective vs. emergency surgery (Table 3).

**Table 3:** Characteristics of the participants with at least one AE (“AE”) and without an AE (“No AE”)

Variable	AE *n* = 131	No AE *n* = 43
Age, years		
Median (Q1, Q3)	82 (76, 86)	77 (74, 84)
Sex		
Female, *n* (%)	76 (58.0)	24 (55.8)
Male, *n* (%)	55 (42.0)	19 (44.2)
Dementia		
Yes, *n* (%)	12 (9.3)	5 (12.5)
No, *n* (%)	117 (90.7)	35 (87.5)
Length of hospital stay (days)		
Median (Q1, Q3)	11 (11,15)	7 (5,11)
Operation time (min)		
Median (Q1, Q3)	82 (50, 146)	47 (25,69)
Length of stay ICU^a^ (h)		
Median (Q1, Q3)	2 (1, 14)	1 (1, 2)
Admission type		
Elective, *n* (%)	63 (48.1)	15 (34.9)
Emergency, *n* (%)	68 (51.9)	28 (65.1)
Level of nursing care ^b^		
Yes, *n* (%)	66 (50.4)	15 (34.9)
No, *n* (%)	65 (49.6)	28 (65.1)
CFS ^c^		
non-frail (< 5), *n* (%)	50 (39.1)	27 (62.8)
frail (≥ 5), *n* (%)	78 (60.9)	16 (37.2)
ASA ^d^		
0, *n* (%)	1 (0.8)	1 (2.4)
1, *n* (%)	15 (11.5)	8 (19.0)
2, *n* (%)	101 (77.1)	31 (73.8)
3, *n* (%)	14 (10.7)	2 (4.8)
ASA-Group		
ASA 1–2, *n* (%)	117 (89.3)	40 (95.2)
ASA 3, *n* (%)	14 (10.7)	2 (4.8)
ISAR^e^		
Median (Q1, Q3)	3 (2,4)	3 (2,3)
BI-prior^f^		
< 50, *n* (%)	11 (8.4)	0
50–80, *n* (%)	34 (26.0)	5 (11.6)
> 80, *n* (%)	86 (65.6)	38 (88.4)
BI-preop^g^		
< 50, *n* (%)	62 (47.3)	14 (32.6)
50–80, *n* (%)	29 (22.1)	16 (37.2)
> 80, *n* (%)	40 (30.5)	13 (30.2)
Living alone/with spouse		
Lives alone, *n* (%)	64 (48.9)	15 (34.9)
With partner/relatives, *n* (%)	66 (50.4)	27 (62.8)
24 h caregiver, *n* (%)	1 (0.8)	1 (2.3)
Living site		
House, *n* (%)	64 (48.9)	26 (60.5)
Apartment, *n* (%)	47 (35.9)	13 (30.2)
Assissted living, *n* (%)	5 (3.8)	1 (2.3)
Nursing home, *n* (%)	15 (11.5)	3 (7)

^a^*ICU* Intensive care unit, ^b^Level of care in Germany “Pflegegrad”, ^c^*CFS* Clinical frailty scale, ^d^*ASA* American Society of Anaesthesiologistˈs-risk classification, ^e^*ISAR* Identification of seniors at risk, ^f^*BI prior* Barthel-Index (BI), which refers to the time 2 weeks prior to hospital admission, ^g^*BI preop* refers to the time preoperatively, ^1^Mann-Whitney-U-Test; ^2^Chi-Quadrat-Test; ^3^Fisher-Exakt-Test; ^4^Fisher-Freeman-Halton-Test

**Fig. 2:**
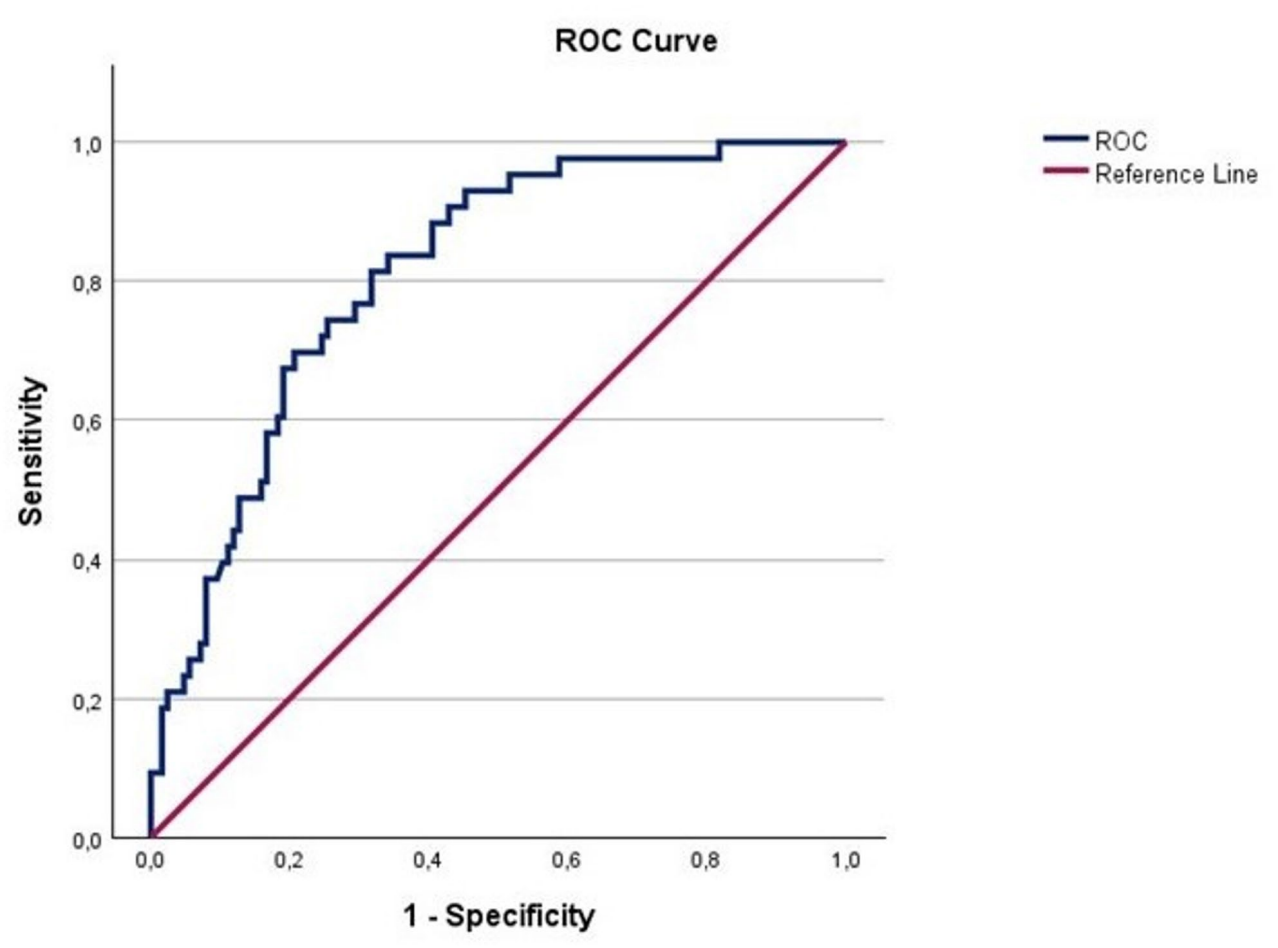
Receiver operating characteristic curve (ROC) of the binary logistic regression model of predicting the absence of AE. AUC 0.810 (CI 0.741; 0.879)

Because we found at least one AE in most of the participants we chose to perform a multivariable binary logistic regression testing for an adverse event-free treatment course. Multivariable binary logistic regression testing showed that the absence of frailty, a shorter operation time and a younger age were associated with an adverse event-free treatment course with an AUC of 0.810 (CI 0.741; 0.879) (Table 4; Fig. 2).

**Table 4:** Results of binary logistic regression for an adverse event-free treatment course, (*n* = 174, 131 (75.3%) AE). Age and operation time were z-transformed, frailty (CFS) was binarized (“frail” and “non-frail”)

Variable	β	SE	*p*	Exp(B)	95% CI for Exp(β)
Age	−0.476	0.226	0.036	0.621	0.399–0.968
Operation time	−1.698	0.382	< 0.001	0.185	0.087–0.390
Frailty (CFS)	−1.167	0.435	0.007	0.311	0.133–0.730

*β* Regression coefficient, *SE* standard error, *p* significance, *Exp(B)* exponential of β, *CI* Confidence interval

## Discussion

Utilizing the IHI GTT during chart review revealed at least one AE in the majority of the study participants. Overall, we found a total of 348 AEs, which relates to 2 AE per admission. In most cases the AE resulted in temporary harm requiring intervention. Intra- and postoperative AE and nosocomial infections were most frequently recorded. Adding a geriatric trigger set was important to identify a significant number of additional AEs.

In contrast to previously published studies, this study reveals a higher incidence of AE [1, 23, 24]. The results observed in other studies were comparable to the outcomes of the current study: A study examined hospitalized patients from all departments aged ≥ 60 years, found 610 AEs in 480 subjects [25]. Of these, 329 (68.5%) had at least one AE. There were 127 AEs per 100 admissions and 22.43 AEs per 1000 patient days. Another study that was conducted in orthopedics with geriatric patients identified 160.7 AE per 100 admissions [8]. The high frequency of AE in this study can be attributed to multiple factors. The high rate of detected AE in our cohort is most certainly driven by (1) a very detailed chart review and modified version of the IHI GTT and (2) the high proportion of older people living with frailty in our study population:

In the original version of the IHI GTT, the review time is limited to 20 min per case [1]. As no time limitations were set in this study, a very detailed chart review was conducted which may have contributed to a higher detection rate of AE as well as to the detection of many AEs not assigned to a trigger. Another reason may be the newly added triggers (Module Geriatrics) and categories (Category C and D) which could have led to the identification of more AE.

The study population had a positive ISAR screening which has been associated with adverse outcomes, such as a prolonged length of stay [26]. Another study showed that the risk of anesthesia-related AE was significantly higher in older surgical patients than in younger individuals [27]. Over half of the participants in our study were older people living with frailty. A study indicated that older people living with frailty have more severe complications and more frequent complications [20, 28]. In colorectal and cardiac surgical patients, older people living with frailty were also more likely to experience complications [29]. This can be confirmed in our study, in the binominal logistic regression a considerable portion of AEs was linked to age and frailty but also operation time (AUC = 0.81), suggesting that an adverse event-free treatment course is more likely with a shorter operation time, as well as non-frail and younger aged patients. The factor with the greatest influence on the occurrence of AE was operation time. This finding is also consistent with the literature in which frailty and prolonged operation time as well as older age were associated with an increased risk of experiencing complications and AEs [19–21, 29–31]. A systematic review and meta-analysis demonstrated that the likelihood of a complication increased by 1% for every additional minute of surgery and by 14% for every 30 min [19]. Considering the fact that the shortage of geriatricians poses a major challenge for the medical care of the aging population, these parameters can be helpful to define more specific criteria by which patients may benefit most from geriatric co-management [32, 33].

In this study we adapted the IHI GTT by adding an additional module “Geriatrics”. Especially the trigger ”New impairment of cognition/vigilance, fluctuating confusion” showed a high PPV and was able to identify the AE delirium in the chart review in 28 cases. Detecting delirium in a geriatric population is crucial because it is frequently underdiagnosed and is a risk factor for complications during hospitalization [5, 34]. The chart review for the newly added triggers associated with infection (“Fever or hypothermia”, “Bacteriuria in urine culture”, “Rising CRP-level”) identified another numerous AE (*n* = 56, 16.1%). Still, these were not sufficient to detect all nosocomial infections, because a further 25 infections (7.1%) were detected without a trigger being recorded. Other AE that where frequently detected without a trigger being recorded were electrolyte disturbances. The IHI White Paper emphasizes the importance of a consistent and standardized application of the IHI GTT to ensure comparability over time or setting [1]. Adding the triggers of the Geriatric module (see Additional file 1) may help to enhance the capability of detecting AEs in a geriatric population but potentially leads to deficits in comparability with other studies using the original version. Further research should evaluate the applicability in other geriatric populations to ensure generalizability of our results, ultimately aiming to improve treatment quality and monitoring of this population with especially high risk for AEs.

### Strength and limitations

The strength of our study is the high proportion of older people living with frailty in our cohort from three surgical departments with emergency as well as elective operations with an ISAR score ≥ 2, potentially close to a real-world population with the option of generalizability. Nevertheless, this heterogeneity of included patients may also count as a limitation. The study gives the opportunity to study relevant AE of this population in great detail. However, because of possible missing documentation in medical records and since further diagnostics would have been necessary in some cases, not all events could be clearly described. Only AE that have been documented by medical staff in enough detail could be considered. I. e. AE that occurred on readmission could only be assessed when the participants were readmitted to Ulm University Medical Center. Readmissions to other hospitals remain unknown. Moreover, due to the difference in sample size, the high rates of AE in GEN and URO are less representative compared to the findings from the TRA department and hinder comparisons among the three departments. Given the relatively small sample size as well as the other limitations mentioned our findings and the adapted GTT should be tested and validated in a broader and more representative study in future research.

## Conclusion

The IHI GTT enabled the identification of a high AE rate in all three surgical settings. The adapted version offers an additional benefit in a geriatric population through the introduction of novel triggers to identify more and relevant AEs.

Younger age, robustness (opposed to frailty), and a shorter operation time were relevant predictors for an AE-free treatment course. Our prediction model offers the opportunity to distinguish high-risk from low-risk individuals for AE development at an early treatment stage which could be helpful to facilitate geriatric co-management.

## Supplementary Information

Supplementary Material 1: Additional file 1. List of all triggers and their definition. 

Supplementary Material 2: Additional file 2. Frequencies of the triggers and the PPV in total and in the three departments

Supplementary Material 3: Additional file 3. Overview of the types of AE and their distribution in total and in the three departments

## Data Availability

Data used in this analysis is derived from the observational and AI development (OKIE) study of the SURGE-Ahead project. Part of the data can be found in the additional files. It can also be accessed on the project’s GitHub page: [https://github.com/IfGF-UUlm/SA\_OKIE](https:/github.com/IfGF-UUlm/SA_OKIE).

## Abbreviations

AE: Adverse Events
ADL: Activities of Daily Living
ASA: American Society of Anesthesiologists
BI: Barthel Index
CFS: Clinical Frailty Scale
CI: Confidence Interval
CRP: C-Reactive Protein
GTT: Global Trigger Tool
HIS: Hospital Information System
IHI: Institute for Healthcare Improvement
ICU: Intensive Care Unit
ISAR: Identification of Seniors at Risk
LOS: Length of Hospital Stay
NCC MERP: National Coordinating Council for Medication Error Reporting and Prevention
NIV: Non-Invasive Ventilation
PACU: Post Anesthesia Care Unit
PPV: Positive Predictive Value
TRA: Trauma Surgery
URO: Urology
GEN: General Surgery
WHO: World Health Organization
