# Supplementary material for: Detection of adverse events in older adults undergoing surgery using the IHI global trigger tool within the SURGE-Ahead project
Source: BMC Geriatr. 2025 Dec 17;26:132. doi: 10.1186/s12877-025-06833-5 (PMC12857066; doi:10.1186/s12877-025-06833-5)
Supplement: Supplementary file 1 — Supplementary Material 1: Additional file 1. List of all triggers and their definition. [file 12877_2025_6833_MOESM1_ESM.docx]

**Additional file 1** List of all triggers and their definition

| Trigger | | Definition |
| --- | --- | --- |
| Modul C care | | |
| C1 | Transfusion of blood or use of blood products | Transfusion of blood products such as erythrocyte concentrates, platelet concentrates, fresh frozen plasma. |
| C2 | Admission to an intensive care unit (ICU) or post anesthesia care unit (PACU) | Postoperative admission of the patient to ICU/PACU |
| C3 | Readmission within 2 days | The readmission to Ulm University Hospital after discharge was considered as a positive trigger. Readmissions to other clinics could not be included in the review. |
| C4 | Readmission within 30 days | Readmission after discharge at Ulm University Hospital. It was not possible to assess readmissions to other clinics. |
| C5 | Arrest/rapid response team | Occurrence of cardiac or pulmonary arrest and/or deployment of the rapid response team. |
| C6 | Unplanned/ acute dialysis | Unexpected need for dialysis. |
| C7 | Ultrasound, X-ray or CT regarding deep vein thrombosis (DVT) and pulmonary embolism (PE). | Ultrasound, X-ray or CT scan if a DVT or PE is suspected. |
| C8 | Patient fall | Patient's fall during hospitalization. |
| C9 | Restraint use | The use of a belt system to restrain the patient if there is a risk of self-harm or harm to others. |
| C10 | In-hospital stroke | Stroke associated with a procedure or anticoagulation |
| C11 | Pressure ulcers | Pressure ulcer occurring during hosptalization |
| C12 | Any procedure complication | An AE associated with any procedure |
| Modul L Laboratory | | |
| L1 | Decrease of greater than 25% in hemoglobin | A decrease in hemoglobin of 25% or more in relation to the initial level. |
| L2 | Glucose <50 mg/dl | Drop in blood glucose level below 50mg/dl. |
| L3 | Positive blood culture^a^ | Detection of pathogens in a blood culture. |
| L4 | Rising BUN or serum creatinine greater than 2 times baseline | An elevation BUN or serum creatinine to a value that is twice the initial value. |
| L5 | International Normalized Ratio (INR) > 6 | Increase in INR > 6. |
| L6 | Partial thromboplastin time (PTT) > 100sec | Increase in PTT > 100 sec. |
| L7 | Post-op troponin level greater than 1.5 ng/ml | Postoperative increase in troponin level above 1.5 ng/ml. |
| L8 | Positive stool culture | Detection of pathogens in a stool sample. In the IHI GTT, only stool cultures with Clostridium difficile are evaluated as triggers. All pathogens are included here. |
| Modul S surgery | | |
| S1 | Re-operation | Re-operation after the first procedure. |
| S2 | Change in procedure | Difference between the surgical method for which information was provided and the surgical method that was actually performed. This also includes intraoperative deviations from the standard procedure. |
| S3 | Intraoperative administration of catecholamines, naloxone, flumazenil | According to the IHI GTT, catecholamines, naloxone and flumazenil are not routinely used intraoperatively and are therefore considered positive triggers when administered. Participants in this study regularly got catecholamines during surgery. Therefore, the administration of catecholamines was only evaluated as a positive trigger if it was documented that an increased administration of catecholamines was necessary. The intraoperative administration of naloxone or flumazenil was evaluated as a positive trigger. |
| S4 | Injury, repair, or removal of organ | Injury, repair, or removal of organ during surgery |
| S5 | Intra-operative death | Especially unexpected intra-operative death. |
| S6 | Any operative complication | AE associated with surgery, including postoperative abnormalities that affect the surgical site (e.g. hematoma, impaired wound healing). |
| Modul I Intensive care | | |
| I1 | (Re-)intubation, tracheotomy, coniotomy, non-invasive ventilation (NIV) | Intubation, tracheotomy, coniotomy or NIV in the ICU. |
| I2 | Mechanical ventilation > 24h postoperative | Mechanical ventilation greater than 24 hours postoperatively. |
| I3 | Readmission to ICU/PACU | Readmission to ICU/PACU during hospitalization |
| I4 | Treatment with procedure in ICU | Every procedure performed in the ICU. |
| I5 | X-ray on ICU/PACU | X-ray imaging in the INT/PACU |
| Modul M Medication | | |
| M1 | Vitamin K administration | Vitamin K administration during hospitalization. |
| M2 | Flumazenil use | Administration of flumazenil as an antidote in the event of a benzodiazepine overdose. |
| M3 | Naloxone use | Administration of naloxone to antagonize opioids. The administration of naloxone in combination preparations (oxycodone/naloxone, tilidine/naloxone) was not included. |
| M4 | Anti-emetics use | Administration of antiemetics during hospitalization. Antiemetics given during anesthesia and in the recovery room are not included. In addition to the IHI GTT, short episodes of nausea and vomiting were also included as an adverse event if this trigger was present (e.g. post-operative nausea and vomiting). |
| M5 | Over-sedation/hypotension | Excessive sedation of the patient due to medication. A reduction in blood pressure may also be an indication of excessive sedation. A drop in the systolic blood pressure ≤ 90 mmHg is considered a positive trigger. If hypotension occurs due to an AE that is not caused by medication, this is also taken into account. |
| M6 | Fenistil/ prednisolone administration | Fenistil/ prednisolone administration during hospitalization. |
| M7 | Abrupt medication stop | Sudden stop of medication. |
| M8 | Other medication problems | Other problems related to medication that are not represented by other triggers. |
| Modul G Geriatrics | | |
| G1 | Bedside watch^a^ | Use of a patient sitter for patients who needs observation (e.g. agitated patients). |
| G2 | New impairment of cognition/vigilance, fluctuating confusion^a^ | Acute changes in cognition/vigilance. Record entries from nurses and physicians using descriptions as “restless, agitated, drowsy, impaired cognition, inattentive, confused” are considered here. This method is also used in other contexts [4,5]. The diagnosis of delirium was made if there was a specific suspicion in the medical record or if it was suspected. |
| G3 | Vomiting^a^ | Vomiting during the hospital stay. Short episodes of vomiting are also included here. |
| G4 | Change in weight >2kg^a^ | Weight gain of more than 2 kg during hospitalization. |
| G5 | Fever or hypothermia^a^ | Body temperature above 38°C or below 36°C. |
| G6 | Bacteriuria in urine culture^a^ | Microbiological detection of bacteria in a urine culture. |
| G7 | Rising CRP-level^a^ | Increase in CRP level during hospital stay > 200 mg/L or no decrease or renewed increase in CRP level on postoperative day 5. |
| Other Trigger | | |
|  | AE without trigger^a^ | All AE that cannot be represented by the triggers used are listed under this trigger. Included AE were:   - Electrolyte disturbances (triggered by medication or other external facors) - Hypo-/hyperkalemia (<3.4, ≥4.6 mmol/l) - Hyper-/hyponatremia (<135, >145 mmol/l) - Hypocalcemia (<2,1 mmol/l) - Nosocomial infection diagnosis - Other |

List of all triggers and their definition. ^a^Newly added triggers. AE: Adverse Event, CT: computer tomography, DVT: Deep vein thrombosis, ICU: Intensive care unit, INR: International Normalized Ratio, NIV: non-invasive ventilation, PACU: Post anesthesia care unit, PE: Pulmonary embolism, PTT: Partial thromboplastin time
