# Supplementary material for: Detection of adverse events in older adults undergoing surgery using the IHI global trigger tool within the SURGE-Ahead project
Source: BMC Geriatr. 2025 Dec 17;26:132. doi: 10.1186/s12877-025-06833-5 (PMC12857066; doi:10.1186/s12877-025-06833-5)
Supplement: Supplementary file 2 — Supplementary Material 2: Additional file 2. Frequencies of the triggers and the PPV in total and in the three departments [file 12877_2025_6833_MOESM2_ESM.docx]

**Additional file 2** Frequencies of the triggers and the PPV in total and in the three departments. The last column presents the number of AE associated with the single trigger.

| **Trigger** | **Total^a^**  **Positive Trigger (n, %)** | **TRA^b^**  **Trigger (n, %)** | **GEN^b^**  **Trigger (n, %)** | **URO^b^**  **Trigger (n, %)** | **Total^c^**  **AE**  **(n, %)** |
| --- | --- | --- | --- | --- | --- |
|  | PPV (%),  (95% CI) | PPV (%),  (95% CI) | PPV (%),  (95% CI) | PPV (%),  (95% CI) |  |
| **Modul C Care** | | | | | |
| **Transfusion of blood or use of blood products** | 58 (10.2) | 45 (11.9) | 5 (5.2) | 8 (8.7) | 47 (11.9) |
|  | 81.0 (71;91) | 80.0 (68;92) | 1/1 AE ident. | 75.0 (45;100) |  |
| **Admission to ICU/PACU** | 50 (8.8) | 20 (5.3) | 14 (14.6) | 16 (17.4) | 21 (5.3) |
|  | 42.0 (28;56) | 70.0 (50;90) | 21.4 (0;43) | 25.0 (4;46) |  |
| **Readmission within 2 days** | 2 (0.4) | 0 | 1 (1.0) | 1 (1.1) | 2(0.5) |
|  | 100 (100;100) | 0 | 1/1 AE ident. | 1/1 AE ident. |  |
| **Readmission within 30 days** | 21 (3.7) | 13 (3.4) | 5 (5.2) | 3 (3.3) | 10 (2.5) |
|  | 47.62 (26;69) | 30.8 (6;56) | 80.0 (44.9;100) | 66.67 (13.3;100) |  |
| **Arrest/rapid response team** | 1 (0.2) | 0 | 0 | 1 (1.1) | 1 (0.3) |
|  | 1/1 AE ident. | 0 | 0 | 1/1 AE ident. |  |
| **Unplanned/ Acute Dialysis** | 0 | 0 | 0 | 0 | 0 |
| **Ultrasound, X-ray or CT for DVT/PE** | 7 (1.2) | 6 (1.6) | 1 (1.0) | 0 | 4 (1.0) |
|  | 57.2 (21;94) | 50.0 (10;90) | 1/1 AE ident. | 0 |  |
| **Documentation of a medical error** | 0 | 0 | 0 | 0 | 0 |
| **Patient fall** | 4 (0.7) | 3 (0.8) | 1 (1.0) | 0 | 3 (0.8) |
|  | 75.0 (33;100) | 66.7 (13;100) | 1/1 AE ident. | 0 |  |
| **Restraint use** | 1 (0.2) | 0 | 1 (1.0) | 0 | 1 (0.3) |
|  | 1/1 AE ident. | 0 | 1/1 AE ident. | 0 |  |
| **In-hospital stroke** | 0 | 0 | 0 | 0 | 0 |
| **Pressure ulcus** | 10 (1.8) | 9 (2.4) | 1 (1.0) | 0 | 10 (2.5) |
|  | 100 (100;100) | 100 (100;100). | 1/1 AE ident. | 0 |  |
| **Any procedure complication** | 1 (0.2) | 0 | 1 (1.0) | 0 | 1 (0.3) |
|  | 1/1 AE ident. | 0 | 1/1 AE ident. | 0 |  |
| **Modul L Laboratory results** | | | | | |
| **Decrease of greater than 25% in hemoglobin** | 62 (10.9) | 45 (11.9) | 11 (11.5) | 6 (6.5) | 59(14.9) |
|  | 95.2 (90;100) | 93.3 (86;100) | 1/1 AE ident. | 1/1 AE ident. |  |
| **Glucose <50 mg/dl** | 1 (0.2) | 1 (0.3) | 0 | 0 | 1 (0.3) |
|  | 1/1 AE ident. | 1/1 AE ident. | 0 | 0 |  |
| **Positive blood culture** | 2 (0.4) | 1 (0.3) | 0 | 1 (1.1) | 1 (0.3) |
|  | 50.0 (0;100) | 1/1 AE ident. | 0 | 0 |  |
| **Rising BUN or serum creatinine greater than 2 times baseline** | 6 (1.1) | 3 (0.8) | 1 (1.0) | 2 (2.2) | 6 (1.5) |
|  | 100 (100;100) | 100 (100;100) | 1/1 AE ident. | 100 (100;100) |  |
| **INR > 6** | 0 | 0 | 0 | 0 | 0 |
| **PTT> 100sec** | 0 | 0 | 0 | 0 | 0 |
| **Post-op troponin level greater than 1.5 ng/ml** | 0 | 0 | 0 | 0 | 0 |
| **Positive stool culture** | 1 (0.2) | 1 (0.3) | 0 | 0 | 1 (0.3) |
|  | 1/1 AE ident. | 1/1 AE ident. | 0 | 0 |  |
| **Modul S Surgery** | | | | | |
| **Re-operation** | 26 (4.6) | 23 (6.1) | 3 (3.1) | 0 | 12 (3.0) |
|  | 46.2 (27-65) | 43.5 (23;64) | 66.7 (13;100) | 0 |  |
| **Change in procedure** | 4 (0.7) | 4 (1.1) | 0 | 0 | 2(0.5) |
|  | 50.0 (1-99) | 50.0 (1;99) | 0 | 0 |  |
| **Intraoperative administration of catecholamines, naloxone, flumazenil** | 3 (0.5) | 2 (0.5) | 0 | 1 (1.1) | 2(0.5) |
|  | 66.7 (13;100) | 50.0 (0;100) | 0 | 1/1 AE ident. |  |
| **Injury, repair, or removal of organ** | 0 | 0 | 0 | 0 | 0 |
| **Any operative complication** | 25 (4.4) | 13 (3.4) | 4 (4.2) | 8 (8.7) | 25  (6.3) |
|  | 100 (100;100) | 100 (100;100) | 100 (100;100) | 100 (100;100) |  |
| **Modul I Intensive Care** | | | | | |
| **Re-/intubation, tracheotomy, coniotomy, NIV** | 5 (0.9) | 3 (0.8) | 2 (2.1) | 0 | 5 (1.3) |
|  | 100 (100;100) | 100 (100;100) | 100 (100;100) | 0 |  |
| **Mechanical ventilation > 24h post-op** | 1 (0.2) | 0 | 1 (1.0) | 0 | 1 (0.3) |
|  | 1/1 AE ident. | 0 | 1/1 AE ident. | 0 |  |
| **Readmission to ICU/PACU** | 4 (0.7) | 1 (0.3) | 2 (2.1) | 1 (1.1) | 4(1.0) |
|  | 100 (100;100) | 1/1 AE ident. | 100 (100;100) | 1/1 AE ident. |  |
| **Treatment with procedure in ICU** | 0 | 0 | 0 | 0 | 0 |
| **X-ray in ICU/PACU** | 9 (1.6) | 7 (1.8) | 2 (2.1) | 0 | 6 (1.5) |
|  | 66.7 (36;98) | 71.4(38;100) | 50.0 (0;100) | 0 |  |
| **Modul M Medication** | | | | | |
| **Vitamin K administration** | 8 (1.4) | 5 (1.3) | 2 (2.1) | 1 (1.1) | 3 (0.8) |
|  | 37.5 (4;71) | 20.0 (0;55) | 50.0 (0;100) | 1/1 AE ident. |  |
| **Flumazenil use** | 0 | 0 | 0 | 0 | 0 |
| **Naloxone use** | 0 | 0 | 0 | 0 | 0 |
| **Anti-emetic use** | 50 (8.8) | 36 (9.5) | 5 (5.2) | 9 (9.8) | 41 (10.4) |
|  | 82.0 (71;93) | 77.8 (64;91) | 80.0 (45;100) | 100 (100;100) |  |
| **Over-sedation/hypotension** | 35 (6.2) | 29 (7.7) | 4 (4.2) | 2 (2.2) | 10 (2.5) |
|  | 28.6 (14;44) | 24.1 (9;40) | 50.0 (1;99) | 50.0 (0;100) |  |
| **Fenistil/prednisolone administration** | 3 (0.5) | 2 (0.5) | 1 (1.0) | 0 | 1 (0.3) |
|  | 33.3 (0;87) | 50.0 (0;100) | 0 | 0 |  |
| **Abrupt medication stop** | 6 (1.1) | 4 (1.1) | 1 (1.0) | 1 (1.1) | 5 (1.3) |
|  | 83.3 (54;100) | 75.0 (33;100) | 1/1 AE ident. | 1/1 AE ident. |  |
| **Other medication problems** | 1 (0.2) | 1 (0.3) | 0 | 0 | 1 (0.3) |
|  | 1/1 AE ident. | 1/1 AE ident. | 0 | 0 |  |
| **Modul G Geriatrics** | | | | | |
| **Bedside watch** | 2 (0.4) | 0 | 0 | 2 (2.2) | 2 (0.5) |
|  | 100 (100;100) | 0 | 0 | 100 (100;100) |  |
| **New impairment of cognition/vigilance, fluctuating confusion** | 36 (6.3) | 26 (6.9) | 5 (5.2) | 5 (5.4) | 30 (7.6) |
|  | 83.3 (71;96) | 84.6 (71;99) | 1/1 AE ident. | 60.0 (17;100) |  |
| **Vomiting** | 32 (5.6) | 22 (5.8) | 5 (5.2) | 5 (5.4) | 21 (5.3) |
|  | 65.6 (49:82) | 59.1 (39;80) | 60.0 (17;100) | 100 (100;100) |  |
| **Change in weight >2kg** | 11 (1.9) | 4 (1.1) | 5 (5.2) | 2 (2.2) | 2 (0.5) |
|  | 18.2 (0;41) | 0 | 40.0 (0;83) | 0 |  |
| **Fever or hypothermia** | 16 (2.8) | 7 (1.8) | 4 (4.2) | 5 (5.4) | 12 (3.0) |
|  | 75.0 (54;96) | 71.4 (38;100) | 75.0 (33;100) | 80.0 (45;100) |  |
| **Bacteriuria in urine culture** | 31 (5.5) | 24 (6.3) | 1 (1.0) | 6 (6.5) | 23 (5.8) |
|  | 74.2 (59;90) | 79.2 (63;95) | 0 | 66.7 (29;100) |  |
| **Rising CRP-level** | 32 (5.6) | 19 (5.0) | 7 (7.3) | 6 (6.5) | 21 (5.3) |
|  | 65.6 (49;82) | 63.2 (45;85) | 57.2 (21;93) | 83.3 (54;100) |  |
| **Total Trigger** | 567 | 379 (66.8) | 96 (16.9) | 92 (16.2) |  |

^a^Percentages refer to the total of 533 positive triggers. ^b^Percentages refer to the total number of triggers in the department. ^c^Percentages refer to 368 AE detected. 1/1 AE ident. = as the trigger was identified only once we did not calculate any PPV or CI. CI: confidence interval, DVT: deep vein thrombosis, ICU: Intensive care unit, INR: International Normalized Ratio, NIV: Non-invasive ventilation, PACU: Post anesthesia care unit, PE: pulmonary embolism, PPV: Positive Predictive Value, PTT: Partial thromboplastin time
