# Supplementary material for: Detection of adverse events in older adults undergoing surgery using the IHI global trigger tool within the SURGE-Ahead project
Source: BMC Geriatr. 2025 Dec 17;26:132. doi: 10.1186/s12877-025-06833-5 (PMC12857066; doi:10.1186/s12877-025-06833-5)
Supplement: Supplementary file 3 — Supplementary Material 3: Additional file 3. Overview of the types of AE and their distribution in total and in the three departments [file 12877_2025_6833_MOESM3_ESM.docx]

**Additional file 3** Overview of the types of AE and their distribution in total and in the three departments

| **Type of AE** | **Total**  **AE (n, %)** | **TRA^a^**  **AE (n, %)** | **GEN^a^**  **AE (n, %)** | **URO^a^**  **AE (n, %)** |
| --- | --- | --- | --- | --- |
| **Intra-/postoperative AE** | 153 (43.8) | 98 (42.2) | 28 (44.4) | 27 (50.0) |
| **Postoperative anemia** | 56 (16.1) | 41 (17.7) | 10 (15.9) | 5 (9.3) |
| **Unexpected intraoperative bleeding** | 13 (3.7) | 7 (3.0) | 2 (3.2) | 4 (7.4) |
| **Postoperative bleeding** | 2 (0.6) | 23 (10.0) | 4 (6.3) | 7 (13.0) |
| **Postoperative vomiting and nausea (PONV)** | 34 (9.8) | 23 (10.0) | 4 (6.3) | 7 (13.0) |
| **Injury during intervention/procedure** | 10(2.9) | 5 (2.2) |  | 5 (9.3) |
| **Other incident during procedure** | 3 (0.9) | 1 (0.4) | 2 (3.2) |  |
| **Change in procedure** | 1 (0.3) |  | 1 (0.4) |  |
| **Hematoma/hematoseroma on surgical site** | 11 (3.2) | 9 (3.9) |  | 2 (3.7) |
| **Anastomotic leak** | 3 (0.9) |  | 2 (3.2) | 1 (1.9) |
| **Displacement of osteosynthesis material** | 4 (1.1) | 4 (1.7) |  |  |
| **Other postoperative AE** | 16 | 6 (2.6) | 7 (11.1) | 3 (5.6) |
| **Nosocomial infection** | 66 (18.9) | 48 (20.7) | 9 (14.3) | 9 (16.7) |
| **Urinary tract infection** | 26 (7.5) | 22 (9.5) |  | 4 (7.4) |
| **Pneumonia** | 9 (2.6) | 9 (3.9) |  |  |
| **Infection of unknown focus** | 12 (3.4) | 5 (2.2) | 2 (3.2) | 5 (9.3) |
| **Wound infection** | 5 (1.4) | 4 (1.7) | 1 (1.6) |  |
| **Surgical site infection** | 5 (1.4) |  | 5 (7.9) |  |
| **Covid-19-infection** | 3 (0.9) | 3 (1.3) |  |  |
| **Other infection** | 6 (1.7) | 5 (2.2) | 1 (1.6) |  |
| **Neurological** | 30 (8.6) | 23 (9.9) | 4 (6.3) | 3 (5.6) |
| **Delirium** | 28 (8.0) | 21 (9.1) | 4 (6.3) | 3 (5.6) |
| **Other** | 2 (0.6) | 2 (0.9) |  |  |
| **Drug side effect** | 18 (5.2) | 14 (6.0) | 4 (6.3) |  |
| **Opioid-induced nausea** | 6 (1.7) | 6 (2.6) |  |  |
| **Hypokalemia/hyponatremia** | 10 (2.9) | 8 (3.5) | 2 (3.2) |  |
| **Other** | 2 (0.6) |  | 2 (3.2) |  |
| **Electrolyte disturbance** | 16 (4.6) | 9 (3.9) | 2 (3.2) | 5 (9.3) |
| **Hypo-/hyperkalemia (<3.4, ≥4.6 mmol/l)** | 9 (2.6) | 6 (2.6) |  | 3 (5.6) |
| **Hyper-/hyponatremia (<135, >145 mmol/l)** | 6 (1.7) | 3 (1.3) | 1 (1.6) | 2 (3.7) |
| **Hypocalcemia (<2,1 mmol/l)** | 1 (0.3) |  | 1 (1.6) |  |
| **Cardiac system** | 15 (4.3) | 9 (3.9) | 3 (4.8) | 3 (5.6) |
| **Arrhythmia** | 7 (2.0) | 5 (2.2) | 1 (1.6) | 1 (1.9) |
| **Cardiac arrest with reanimation** | 1 (0.3) |  |  | 1 (1.9) |
| **Heart attack, Angina pectoris** | 2 (0.6) | 1 (0.4) |  | 1 (1.9) |
| **Hydropic/cardiac/cardiopulmonary decompensation** | 4 (1.1) | 2 (0.9) | 2 (3.2) |  |
| **Hypertensive deregulation** | 1 (0.3) | 1 (0.4) |  |  |
| **Pulmonary system** | 15 (4.3) | 8 (3.4) | 6 (9.5) | 1 (1.9) |
| **Acute respiratory insufficiency** | 9 (2.6) | 5 (2.2) | 4 (6.3) |  |
| **Pleural effusion** | 3 (0.9) | 1 (0.4) | 1 (1.6) | 1 (1.9) |
| **Other** | 3 (0.9) | 2 (0.9) | 1 (1.6) |  |
| **Renal system** | 11 (3.2) | 7 (3.0) | 2 (3.2) | 2 (3.7) |
| **Acute kidney injury** | 8 (2.3) | 6 (2.6) | 1 (1.6) | 1 (1.9) |
| **Other** | 3 (0.9) | 1 (0.4) | 1 (1.6) | 1 (1.9) |
| **Nursing care** | 10 (2.9) | 9 (3.9) | 1 (1.6) |  |
| **Pressure ulcer (newly developed)** | 9 (2.6) | 9 (3.9) |  |  |
| **Pressure ulcer (deterioration)** | 1 (0.3) |  | 1 (1.6) |  |
| **Gastrointestinal system** | 9 (2.6) | 2 (0.9) | 3 (4.8) | 4 (7.4) |
| **Sub-/Ileus** | 3 (0.9) |  | 1 (1.6) | 2 (3.7) |
| **Acute abdomen** | 2 (0.6) |  |  | 2 (3.7) |
| **Gastrointestinal bleeding** | 3 (0.9) | 1 (0.4) | 2 (3.2) |  |
| **Liver failure** | 1 (0.3) | 1 (0.4) |  |  |
| **Allergic reaction** | 3 (0.9) | 3 (1.3) |  |  |
| **Drug allergy** | 2 (0.6) | 2 (0.9) |  |  |
| **Allergic contact dermatitis** | 1 (0.3) | 1 (0.4) |  |  |
| **Other** | 2 (0.6) | 1 (0.4) | 1 (1.6) |  |
| **Hyperglycemia > 400 mg/dl** | 1 (0.3) | 1 (0.4) |  |  |
| **Hypotension** | 1 (0.3) |  | 1 (0.3) |  |
| **Total** | 348 (100) | 231 (66.4) | 63 (18.1) | 54 (15.5) |

^a^Percentages refer to the total number of AE in the respective department. AE: Adverse event, PONV: Postoperative vomiting and nausea.
